# COVID-19 lockdowns weigh heavily on youth: an analysis of the impact on BMI for Age Z scores in children and adolescents

**DOI:** 10.1093/pubmed/fdad287

**Published:** 2024-01-30

**Authors:** Muna Abed Alah, Sami Abdeen, Iheb Bougmiza, Nagah Selim

**Affiliations:** Community Medicine Department, Hamad Medical Corporation (HMC), Doha, Qatar; Community Medicine Department, Hamad Medical Corporation (HMC), Doha, Qatar; Community Medicine Department, Primary Health Care Corporation (PHCC), Doha, Qatar; Community Medicine Department, College of Medicine, Sousse University, Sousse, Tunisia; Community Medicine Department, Primary Health Care Corporation (PHCC), Doha, Qatar; Public Health and Preventive Medicine, Cairo University, Cairo, Egypt

**Keywords:** BAZ, children, COVID-19, lifestyle, obesity, school closures, students

## Abstract

**Background:**

The COVID-19 pandemic has significantly impacted the lifestyle and health of children and adolescents. This study aimed to assess the lifestyle changes brought about by COVID-19-related school closures and their impact on the Body Mass Index for Age Z (BAZ) scores of governmental school students in Qatar.

**Methods:**

An analytical cross-sectional study was conducted between June and August 2022 targeting students aged 8–15 years. Data on lifestyle behaviors were gathered through telephone interviews with parents of selected students. The BAZ scores before and after school closures that were automatically calculated by the electronic health records system were extracted.

**Results:**

We completed 1546 interviews. We found a significant increase in unhealthy food categories, a reduction in physical activity and an increase in the screen time over the period of schools’ closure. The BAZ increased significantly by 0.30 (95% CI 0.26–0.35). The increase in BAZ scores was significantly higher among male students and the younger age group compared to females and older counterparts, respectively. The student’s age group, sex, nationality and change in physical activity were significant predictors of the change in BAZ scores.

**Conclusions:**

School closures during the COVID-19 pandemic negatively impacted the lifestyle of students in Qatar and resulted in a significant increase in the BAZ scores.

## Introduction

As evidence continues to mount, children and adolescents are reportedly experiencing tremendous adverse health effects from the COVID-19 pandemic. The closure of schools and movement restrictions helped limit the spread of the infection, yet it negatively impacted the lifestyle of many around the world.^1^^,^^2^ Adverse dietary changes, reduction in physical activity and more sedentary behaviors have been reported among children and adolescents during COVID-19-related school closures.^3–7^ Such changes can contribute to increased body weight and accordingly body mass index (BMI). A study in Spain observed that children’s daily screen time rose by an average of 2.9 h.^7^ Meanwhile, in Italy, the percentage of children and adolescents engaging in over 4 h of smartphone use daily surged from 16.3% pre-lockdown to 66.3% during the lockdown period.^8^ Research examining changes in physical activity among young people across various countries in Latin America (Brazil, Chile, Colombia) and Europe (Spain, Italy) found a rise in the percentage of inactive adolescents, from 73.0% pre-lockdown to 79.5% during lockdown.^9^ In the Middle East, studies from Saudi Arabia and Egypt revealed dietary challenges among children and adolescents during the pandemic. In Saudi Arabia, 39.4% reported increased consumption of simple carbohydrates, fried foods and soft drinks,^10^ while in Egypt, 45.6 and 37.6% noted a rise in sweets, unhealthy foods and more frequent snacking post-COVID-19 closures.^11^

A report by the Centers for Disease Control and Prevention (CDC) highlighted that during the COVID-19 pandemic, the monthly BMI increase rate among children and adolescents aged 2–19 years doubled compared to the pre-pandemic period (0.100 kg/m^2^ versus 0.052 kg/m^2^).^12^ A recently published systematic review and meta-analysis showed a significant body weight gain during the lockdown and a substantial increase in BMI among children and adolescents in 12 studies.^13^ In Jordan, a study conducted among children and adolescents showed a significant increase in the mean BMI for Age Z scores (BAZ) that increased from 0.32 ± 1.9 before the pandemic to 0.82 ± 1.9 during the lockdown among children, and from 0.35 ± 1.43 before lockdown to 0.54 ± 1.47 among adolescents.^6^ Consuming unhealthy diets such as junk food, fried food and sugar-sweetened beverages can lead to obesity.^14^ Several adverse metabolic outcomes including metabolic syndrome, decreased fitness and unfavorable body composition can result from sedentary behaviors and reduced physical activity.^15–21^ Moreover, these unhealthy behaviors are associated with decreased academic achievement^22^ and impaired psychological health.^23^ Adverse physiological, psychological and educational well-being outcomes were also reported in the literature as consequences of excessive screen time.^24^

As a step to contain the spread of COVID-19, and to ensure the safety of all students and school staff, Qatar imposed the closure of schools that started in March 2020. The schools went through different phases of closure ranging from complete to partial closure (with blended learning). In September 2021, schools returned to the normal state with complete face-to-face learning.^25^

In this study, we aimed to assess the adverse lifestyle changes (in diet, physical activity and screen time) brought about by COVID-19-related school closures and their impact on the BAZ scores of children and adolescents at government schools in Qatar. To the best of our knowledge, studies linking the parent-reported data with actual body measurements of children are limited.^6^^,^^25^ This study is the first of its kind in Qatar and is part of a larger national project that assessed the impact of COVID-19-related school closures on the lifestyle and vision of children and adolescents. Addressing the impact of lifestyle changes on BAZ scores during the pandemic is crucial as it provides insights into the direct consequences of altered daily routines on children’s health. The pandemic-induced shift in lifestyle, marked by increased sedentary behavior and dietary changes, potentially disrupts normal growth patterns in children. Understanding these impacts is essential for developing targeted interventions to mitigate long-term health risks associated with these unprecedented lifestyle shifts.

## Methods

An analytical cross-sectional study targeting students from the 3rd to 9th grades at governmental schools was conducted between June and August 2022 including all nationalities.

### Study procedure and sampling technique

A range of preventive and clinical health services are provided through school health clinics that are operated by highly qualified trained school nurses under the umbrella of the School Health Services and Programs department of primary health care corporation (PHCC), at all government schools of all educational levels (primary, preparatory and secondary).^26^ Growth monitoring is one of the services done by the department, which includes measuring weight, height and BMI of all students annually. All the collected measurements are entered directly into the electronic health records system.

We retrieved a list of all government schools’ students between the 3rd and the 9th grades and stratified them by sex and age (8–11 years, 12–15 years) into four strata from the national electronic health records system in Qatar. Then, using a stratified sampling technique, we randomly selected a proportionate number of students from each stratum. We collected the primary data by telephone interviews with the parents of selected students, while the growth measurements were retrieved from the electronic health records system. Verbal consent was obtained and documented for each participant.

### Data collection

A questionnaire was adapted from multiple tools in English and translated into Arabic by an accredited translation body (see Supplementary Material 1 and Material 2). We assured the translational, face and content validities by distributing the questionnaire to experts in the field who rated the different items. The scale content validity index using the universal agreement approach S-CVI (UA) for the diet items was 0.83, and 0.95 using the average approach, indicating acceptable content validity. The questionnaire was piloted on a convenience sample who were excluded from the final sample.

### The data collection tool

In the first section of the questionnaire, we addressed the sociodemographic characteristics and background information of students such as their age, nationality, age of their mothers and fathers, their school grade, the highest educational level of their mothers and fathers, mother’s employment status and other general information. The changes in dietary behaviors and physical activity before and during school closures were assessed in the second and third sections, respectively. We adapted these sections from existing validated short-form survey instruments for children’s diet, physical activity and sedentary behaviors developed based on the recommendations of the Sax Institute.^27^^,^^28^ In the last section, we addressed the changes in screen time.

### Outcome measures

The assessment methods for changes in diet and physical activity were previously described in detail.^29^ To assess the change in screen time, parents were asked to indicate their children’s average daily screen time in hours during weekdays and weekends before and during the closure. The difference between these two values indicated the change in screen time. BAZ scores were extracted from the electronic health records system at two time points: before school closures (between September 2019 and March 2020) and after the closure (between September 2021 and March 2022). The change in BAZ was calculated by subtracting the BAZ before the school closures from the BAZ after the closures for each student. The BAZ score is a measure of relative BMI adjusted for a child’s age and sex, compared to a reference standard—in this case, the World Health Organization BMI-for-age growth charts.^30^ This measurement is crucial for children as their bodies naturally gain BMI as they develop and mature. A BAZ change of 0 suggests that a child has maintained a consistent BMI over time, compared to other children of the same age and sex during the same period. Conversely, an increase in BAZ scores indicates a more rapid gain in BMI, while a negative change suggests a less rapid gain in BMI compared to the reference sample of children of the same age and sex.^31^

### Statistical analysis

IBM SPSS Statistics for Windows^32^ was used to analyze the data. Categorical data were summarized using percentages while the numerical data were summarized using mean and standard deviation. For dichotomous variables, comprising only two categories, the *t*-test was utilized to compare BAZ scores and the changes in BAZ scores. In contrast, for variables with more than two categories, analysis of variance (ANOVA) was applied. To assess the significance of the changes in diet, physical activity, screen time and BAZ over the period of school closures, the Wilcoxon signed-rank test or the paired Student’s *t*-test was used as indicated. A linear regression was executed to explore the potential predictors of the changes in BAZ. The selection of independent variables for the linear regression model was a multifaceted process, combining statistical evidence, literature review and theoretical considerations. Variables with *P*-values <0.25 in the univariate analysis were initially considered to capture potential predictors that might not meet the stricter conventional significance levels. In addition, variables identified in existing literature as significant predictors of Body BAZ changes, as well as those theoretically deemed relevant, including some not extensively studied before were included. The associations between risk factors and outcomes were presented as adjusted regression coefficient (beta) and 95% confidence intervals (95% CIs). *P*-values less than 0.05 were considered significant.

### Ethical considerations

The study has been ethically approved by the Institutional Review Board of Hamad Medical Corporation (MRC-03-21-895).

## Results

### The sociodemographic characteristics of students and other background information

Of the 3327 parents contacted for telephone interviews regarding their children aged 8 to 15 years, 322 (9.7%) explicitly refused to participate and 1459 (43.9%) did not answer, resulting in 1546 completed questionnaires and a response rate of ~46%. In cases where information on the age of either the father or mother was unavailable, often due to the unfortunate circumstance of one parent being deceased, we encountered missing data. However, as these instances constituted less than 5% of our total data, we did not make any specific adjustments for these missing values. The mean age of the students was 11 ± 2 distributed as 845 (54.7%) between 8 and 11 years of age, and the remaining between 12 and 15 years. Males and females were roughly equally represented with 50.3% female students. Most of the students were Arabs (89.1%). A positive family history of obesity or overweight in one or more of the first-degree relatives of the child was found in 38% of the students, while 8% had chronic diseases. About half of the mothers and fathers had a college degree or higher. We found that 46% of mothers were employed (Table 1).

**Table 1:** Sociodemographic characteristics and health-related information of included students

**Characteristic**		***N* = 1546 No (%)**
**Student age (M ± SD)**		11 ± 2
**Student’s age categories**	8–11 years	845 (54.7)
	12–15 years	701 (45.3)
**School stage**	Primary school (3rd to 6th grades)	973 (62.9)
	Preparatory school (7th to 9th grades)	573 (37.1)
**Gender**	Female	777 (50.3)
	Male	769 (49.7)
**Nationality** ^*^	Non-Qatari	974 (63.0)
	Qatari	572 (37.0)
**Ethnicity**	Non-Arab	169 (10.9)
	Arab	1377 (89.1)
**Number of siblings**	3 or less	740 (47.9)
	4–6	655 (42.4)
	>6	151 (9.8)
**Family income**	Less than 10 000 QR	189 (12.2)
	10 000–30 000 QR	278 (18.0)
	30 000–50 000 QR	48 (3.1)
	More than 50 000 QR	35 (2.3)
	Don’t want to answer	996 (64.4)
**Mother’s education**	No formal education	76 (4.9)
	Primary school level	101 (6.5)
	Preparatory school level	126 (8.2)
	Secondary/high school level	466 (30.1)
	College or higher	777 (50.3)
**Father’s education**	No formal education	40 (2.6)
	Primary school level	77 (5.0)
	Preparatory school level	137 (8.9)
	Secondary/high school level	414 (26.8)
	College or higher	878 (56.8)
**Mother’s employment**	Employed	711 (46.0)
	Not employed	835 (54.0)
**Health-related information**		
**Chronic disease** ^†^	No	1423 (92.0)
	Yes	123 (8.0)
**Family history of obesity**	No	958 (62.0)
	Yes	588 (38.0)

*Abbreviations*: M, mean; SD, standard deviation

^†^Most commonly reported chronic disease was asthma

^^*^^28 different nationalities were reported

### Dietary changes during COVID-19-related school closures

Most students consumed one to two servings of fruit and one to three servings of vegetables per day before and during closure. About 24% of students were consuming less than one serving/day of fruit before schools’ closure and this percentage increased to ~28% during the closure, while 7.4% increased to a higher intake category. On the other hand, the parents of 84 (5.4%) students reported a reduction in their children’s vegetable intake to a lower category during school closures. Overall, we found a nonsignificant increase in the intake of fruit during closure and a significant decrease in the intake of vegetables (*P* < 0.001).

The frequency of sweetened beverages was ~1–3 cups/week before and during closure. About 22% of students were consuming less than one cup/week before the closure, and this proportion was further decreased to 18.7% during closure. Approximately 16% of the students increased their intake to higher amount categories.

Most of the students were consuming fried food prepared at home, and junk food on a frequency of one to two times/week before and during the closure. The proportion of those consuming this food three to four times/week increased by 12 (for fried food) and 11% (for junk food) during the closure.

The frequency of the intake of sugar-based products and sweets (candies, chocolate, jam…) was mostly on an average of one to two times/week before the closure and three to four times/week during the closure. The percentage of students who were consuming such food less than once/week dropped to 5% from 7.8%, while the percentage of those consuming this food two times or more/day more than doubled during schools’ closure.

Overall, we found significant increases in the frequency of intake of sweetened beverages (*P* < 0.001), fried food prepared at home (*P* < 0.001), junk food (*P* = 0.035) and sweets (*P* < 0.001) during the closure compared to before (Table 2).

**Table 2:** The changes in diet and physical activity over the period of schools’ closure in the total sample using Wilcoxon signed-rank test

**Variable**		***N***	***Mean rank***	***Sum of ranks***	**Direction of change**	***P*-value** ^*^	***r***
**Fruit intake**	Negative ranks	104	109	11 336	Increased	0.459	—
	Positive ranks	114	110	12 535			
**Vegetable intake**	Negative ranks	84	63	5292	Decreased	**<0.001**	−0.3
	Positive ranks	41	63	2583			
**Soft drink intake**	Negative ranks	146	224	32 711	Increased	**0.021**	0.1
	Positive ranks	241	176	42 366			
**Home fried food intake**	Negative ranks	38	236	8974.5	Increased	**<0.001**	0.8
	Positive ranks	376	205	76 930.5			
**Junk food intake**	Negative ranks	209	345	72 120.5	Increased	**0.035**	0.1
	Positive ranks	356	247	87 774.5			
**Sweets intake**	Negative ranks	40	299	11 969.5	Increased	**<0.001**	0.8
	Positive ranks	524	281	147 360.5			
**Physical activity**	Negative ranks	736	402	296 133	Decreased	**<0.001**	−0.7
	Positive ranks	96	525	50 395			

*Abbreviation*: *r*, rank-biserial correlation coefficient.

^*^Negative ranks indicate decreased frequency during closure and positive ranks indicate increased frequency during closure

### Changes in physical activity during COVID-19-related school closures

Most of the students (66.8%) used to practice physical activity on an average of 2–4 days/week before closure, and this percentage reduced by half during closure to 32%. On the other hand, the proportion of those practicing physical activity on one day or less increased from ~15% before to 52% during closure. The reduction in physical activity during school closures was significant in the total sample (Table 2), in different sexes and in different age groups (*P* < 0.001).

### Changes in screen time during COVID-19-related schools’ closures

Excluding the time spent attending online classes, the mean screen time for the whole week before schools’ closure was found to be 17.4 ± 10.5 h (2.5 ± 1.5 h/day), and 28.9 ± 14.1 h/week (4.1 ± 2.0 h/day) during closure. A significant increase in screen time by 11.5 ± 11.6 h across the week was reported (*P* < 0.001) (Table 3). The mean screen time during a weekday was 2.3 ± 1.5 h, and 3.0 ± 2.1 h during a weekend day before closure, which increased to 4.1 ± 2.1 h, and 4.3 ± 2.2 h during the closure, respectively.

**Table 3:** The changes in screen time and BAZ scores over the period of COVID-19-related schools’ closure among children and adolescents in Qatar using paired Student’s *t*-test

**Screen time (h)**		**M ± SD**	**Change** **M ± SD**	**Direction of change**	***P*-values**
**All week (h/week)**	**Before school closure**	17.4 ± 10.5	11.5 ± 11.6	Increased	**<0.001**
	**During school closure**	28.9 ± 14.1			
**In a typical weekday (h/day)**	**Before school closure**	2.5 ± 1.5	1.6 ± 1.7	Increased	**<0.001**
	**During school closure**	4.1 ± 2.0			
**On weekdays (h/day)**	**Before school closure**	2.3 ± 1.5	1.8 ± 1.9	Increased	**<0.001**
	**During school closure**	4.1 ± 2.1			
**On weekends (h/day)**	**Before school closure**	3.0 ± 2.1	1.3 ± 1.7	Increased	**<0.001**
	**During school closure**	4.3 ± 2.2			
**BAZ scores**	**Before school closure**	0.6 ± 1.6	0.30 ± 0.89	Increased	**<0.001**
	**After school closure**	0.9 ± 1.6			

*Abbreviations*: M, mean; SD, standard deviation

### The impact of COVID-19-related schools’ closure on the BAZ scores of students

**Fig. 1:**
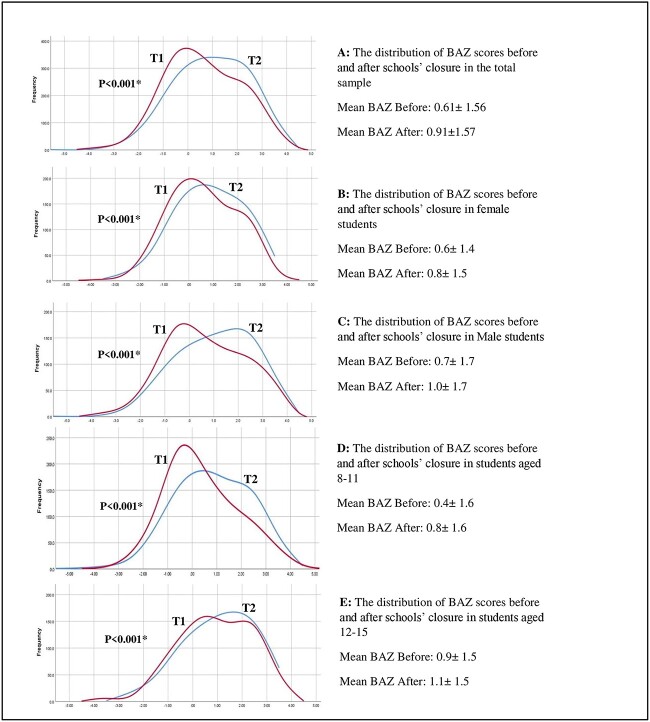
The distribution of BAZ scores before and after schools’ closure in the total sample (**A**), both sexes (**B**, **C**) and age groups (**D**, **E**). The distribution of BAZ scores before (T1) was shifted to the right after closure (T2) in the total sample and the different subgroups indicating an increase in the mean BAZ. ^*^P-value indicating significant increases in the mean BAZ over the period of school closures using paired Students’ *t*-test.

The mean BAZ before school closures was found to be 0.61 ± 1.56 in the total sample, and 0.91 ± 1.57 after closure (as measured after the reopening of schools in the academic year 2021–2022), representing a significant increase of 0.30 during the period of school closures (*P* < 0.001) as shown in Table 2. It increased by 0.25 (95% CI 0.20–0.31, *P* < 0.001) among females, and by 0.35 (95% CI 0.28–0.42, *P* < 0.001) among males. Similarly, significant increases were found among those 12–15 years by 0.15 (95% CI 0.09–0.20, *P* < 0.001), and among those 8–11 years by 0.43 (95% CI 0.37–0.49, *P* < 0.001) as shown in Fig. 1. Using the independent *t*-test to compare the change (increase) in BAZ across different subgroups, we found that the increase was significantly higher among males compared to females (*P* = 0.028), and among those 8–11 years of age compared to the older age group (*P* < 0.001).

**Fig. 2:**
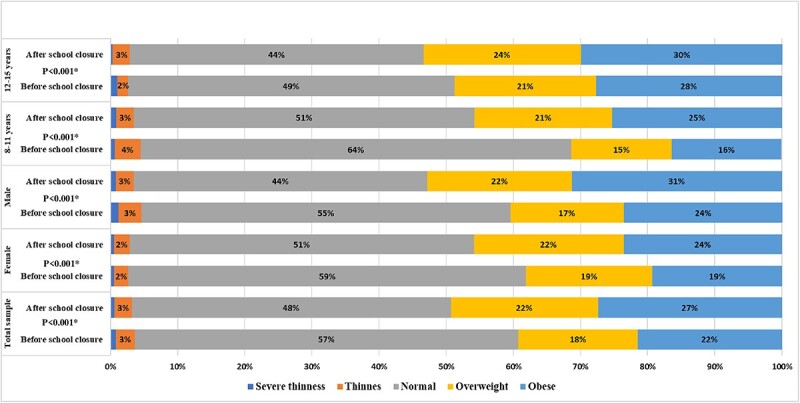
The proportion of students in each BMI for age category before and after schools’ closure in the total sample, both sexes and age groups. The proportions of students with severe thinness were less than 1%. ^*^P-values indicating significant increases in the proportion of students falling in a higher BMI for age categories using the Wilcoxon matched pair signed-rank test.

Before the closure, the prevalence of obesity (defined as BAZ >2 or having a BMI for age that falls more than 2 SD above the median) was found to be 21.5%, which increased significantly to 27.4% after school closures according to the BAZ measurements taken after the reopening of schools in 2021–2022 (*P* < 0.001). The prevalence of obesity also increased significantly among females (from 19.3 to 23.6%, *P* < 0.001), males (from 23.7 to 31.3%, *P* < 0.001) and those 8–11 years (from 16.3 to 25.3%, *P* < 0.001). However, the increase in the prevalence of obesity among those 12–15 years was not significant. Of the total sample, 17.8% were overweight (defined as BAZ >1 and ≤2 or having a BMI for age that falls between 1 and 2 SD above the median) before schools’ closure. The prevalence increased to 21.9% according to the measurements taken after reopening of schools in 2021–2022. In summary, almost 50% of the included children and adolescents were found to be obese or overweight after the reopening of schools. Using the Wilcoxon matched pair signed-rank test, we found a significantly higher proportion of children and adolescents falling in higher BMI for age categories after schools’ closure compared to before (*P* < 0.001) as shown in Fig. 2.

### Determinants and predictors of the change in BAZ scores of students

The univariable analysis showed that the mean increase in BAZ score was significantly higher among younger (*P* < 0.001), males (*P* = 0.028) and Qatari students (*P* = 0.008), whose fathers aged <35 years (*P* = 0.010) and whose mothers were employed (*P* = 0.048) compared with others. No significant associations were found between the change in BAZ scores and the changes in diet, physical activity and screen time (Table 4).

We determined the predictors of the change (difference) in BAZ scores using linear regression. The Omnibus Test, which evaluates the overall significance of the regression model, yielded a *χ*^2^ = 175.022, with a *P*-value of <0.001, indicating that the model is statistically significant. The adjusted *R*^2^ was 0.099.

To ensure the robustness and validity of our regression analysis, we rigorously tested the key assumptions. The linearity assumption was assessed using a scatter plot for the baseline BAZ score (the only continuous independent variable used in the model) against the dependent variable showing linear relationship. For categorical variables, linearity was assumed. Homoscedasticity was confirmed through the Breusch–Pagan test, yielding a *P*-value of 0.091, suggesting that the assumption of homoscedasticity was not violated at the conventional significance levels. Normality of residuals was assessed visually using histograms, indicating an approximate normal distribution. To assess autocorrelation in the residuals, the Durbin–Watson statistic was utilized, yielding a value of 2.065. This falls within the acceptable range of 1.5 to 2.5, indicating no significant autocorrelation concerns in our model. Multicollinearity was examined using the variance inflation factor (VIF), with results showing a minimum VIF of 1.05 and a maximum of 1.83. These values are well below the commonly used threshold of 10 (or 5 in more conservative analyses), indicating no multicollinearity issues among the independent variables.

After adjusting for baseline BAZ, the model showed that the student’s age group, sex, nationality and the change in physical activity were significant predictors of the change in BAZ scores as shown in Table 5. Being a younger (8–11 years), male student, of Qatari nationality and with reduced physical activity was associated with a 0.21 (95% CI 0.13–0.30, *P* < 0.001), 0.10 (95% CI 0.02–0.19, *P* = 0.019), 0.12 (95% CI 0.03–0.21, *P* = 0.009) and 0.13 (95% CI 0.04–0.21, *P* = 0.004) increase in the BAZ difference compared to older, female students, non-Qatari and those who did not decrease their physical activity, respectively.

**Table 4:** Bivariate analysis of the change in BAZ scores of students over the period of COVID-19-related school closures among different subgroups using Student’s *t*-test or ANOVA

**Characteristics**		**Change in BAZ scores**	
		**M ± SD**	***P*-value**
**Student age categories**	8–11 years	0.43 ± 0.92	**<0.001**
	12–15 years	0.15 ± 0.78	
**Sex**	Female	0.25 ± 0.78	**0.028**
	Male	0.35 ± 0.95	
**Nationality (Qatari, non-Qatari)**	Non-Qatari	0.26 ± 0.85	**0.008**
	Qatari	0.38 ± 0.91	
**Number of siblings**	3 or less	0.30 ± 0.94	0.713
	4–6	0.29 ± 0.82	
	>6	0.36 ± 0.78	
**Chronic diseases**	No	0.30 ± 0.87	0.663
	Yes	0.34 ± 0.92	
**Family history of obesity**	No	0.29 ± 0.88	0.620
	Yes	0.32 ± 0.86	
**Mother age categories**	<35	0.35 ± 0.83	0.613
	35–44	0.29 ± 0.87	
	45–54	0.30 ± 0.91	
	55 or more	0.04 ± 0.90	
**Father age categories**	<35	0.61 ± 0.88	**0.010**
	35–44	0.31 ± 0.83	
	45–54	0.24 ± 0.92	
	55 or more	0.30 ± 0.84	
**Mother education**	No formal education	0.39 ± 0.90	0.414
	Primary school level	0.27 ± 0.82	
	Preparatory school level	0.32 ± 1.06	
	Secondary/high school level	0.35 ± 0.90	
	College or higher	0.26 ± 0.83	
**Father education**	No formal education	0.51 ± 0.78	0.593
	Primary school level	0.34 ± 0.79	
	Preparatory school level	0.26 ± 0.92	
	Secondary/high school level	0.31 ± 0.95	
	College or higher	0.29 ± 0.84	
**Mother employment**	Employed	0.35 ± 0.93	**0.048**
	Not employed	0.29 ± 0.81	
**Change in fruit intake**	No decrease	0.30 ± 0.86	0.744
	Decrease	0.28 ± 1.00	
**Change in vegetable intake**	No decrease	0.31 ± 0.86	0.321
	Decrease	0.21 ± 1.03	
**Change in sweetened beverage intake**	No increase	0.32 ± 0.85	0.062
	Increase	0.20 ± 0.98	
**Change in home fried food intake**	No increase	0.33 ± 0.87	0.062
	Increase	0.23 ± 0.87	
**Change in junk food intake**	No increase	0.30 ± 0.83	0.974
	Increase	0.30 ± 0.99	
**Change in sugar-based products and sweets intake**	No increase	0.30 ± 0.83	0.995
	Increase	0.30 ± 0.95	
**Change in physical activity**	No decrease	0.30 ± 0.89	0.858
	Decrease	0.31 ± 0.86	
**Change in screen time**	No increase	0.33 ± 0.85	0.421
	Increase	0.29 ± 0.88	

*Abbreviations*: M, mean; SD, standard deviation

**Table 5:** Multivariable linear regression analysis for the predictors of the change in BAZ scores of students over the period of COVID-19-related school closures

**Characteristics**		**Adjusted *β* (unstandardized) (95% CI)**	*P*-value^*^^*^
**Student age categories**	8–11 years	0.21 (0.13–0.30)	**<0.001**
	12–15 years	1 [Reference]	
**Sex**	Female	−0.10 (−0.19 to −0.02)	**0.019**
	Male	1 [Reference]	
**Nationality (Qatari, non-Qatari)**	Non-Qatari	−0.12 (−0.21 to −0.03)	**0.009**
	Qatari	1 [Reference]	
**Number of siblings**	3 or less	—	—
	4–6	—	—
	>6	—	—
**Chronic diseases**	No	—	—
	Yes	—	—
**Family history of obesity**	No	—	—
	Yes	—	—
**Mother age categories**	<35	—	—
	35–44	—	—
	45–54	—	—
	55 or more	—	—
**Father age categories**	<35	1 [Reference]	
	35–44	−0.17 (−0.38 to 0.04)	0.109
	45–54	−0.20 (−0.42 to 0.01)	0.058
	55 or more	−0.10 (−0.34 to 0.13)	0.384
**Mother education**	No formal education	—	—
	Primary school level	—	—
	Preparatory school level	—	—
	Secondary/high school level	—	—
	College or higher	—	—
**Father education**	No formal education	—	—
	Primary school level	—	—
	Preparatory school level	—	—
	Secondary/high school level	—	—
	College or higher	—	—
**Mother employment**	Employed	0.08 (−0.01 to 0.17)	0.069
	Not employed	1 [Reference]	
**Change in fruit intake**	No decrease	1 [Reference]	
	Decrease	0.09 (−0.14 to 0.32)	0.442
**Change in vegetable intake**	No decrease	1 [Reference]	
	Decrease	−0.05 (−0.31 to 0.20)	0.686
**Change in sweetened beverage intake**	No increase	1 [Reference]	
	Increase	−0.12 (−0.24 to 0.01)	0.074
**Change in home fried food intake**	No increase	1 [Reference]	
	Increase	−0.09 (−0.20 to 0.02)	0.120
**Change in junk food intake**	No increase	1 [Reference]	
	Increase	0.07 (−0.05 to 0.18)	0.273
**Change in sugar-based products and sweets intake**	No increase	1 [Reference]	
	Increase	−0.03 (−0.14 to 0.07)	0.530
**Change in physical activity**	No decrease	1 [Reference]	
	Decrease	0.13 (0.04–0.21)	**0.004**
**Change in screen time**	No increase	1 [Reference]	
	Increase	−0.03 (−0.13 to 0.07)	0.550

*Abbreviation*: CI, confidence interval

## Discussion

There is a growing body of literature that illustrates the negative impact of the restrictive measures imposed during the COVID-19 pandemic on the lifestyle and health of children and adolescents. This study aimed to assess the adverse lifestyle changes (in diet, physical activity and screen time) caused by COVID-19-related school closures and their impact on the BAZ scores among this crucial segment of the population.

### Main findings of the study

In this study, we found a significant increase in unhealthy dietary items such as sweetened beverages, fried food and junk food, a significant reduction in vegetable intake and in physical activity, and significant increases in screen time and BAZ scores over the period of school closures.

### What is already known on this topic

The movement restrictions during the lockdown measures affected food-related practices. Many people tended to stay at home, limited their grocery shopping, decreased their intake of fresh food and depended more on long-lasting, easier-to-store shelf-stable alternatives, which tend to be ultra-processed.^2^ Consequently, this might have affected children’s dietary patterns and behavior since parents are responsible for buying and preparing food for their children, whose intake of food is influenced by what is available in the house. Though not investigated in this study, parental lifestyle behaviors greatly influence those of their children, which can be critically important considering the negative impact of COVID-19 on the lifestyle of adults spotted in the literature.^1^^,^^2^^,^^33–35^ We found a significant reduction in vegetable intake and a significant increase in unhealthy food like sweetened beverages, fried food, fast food and sugar-based products and sweets during the school closures corroborating the results of previous studies.^6^^,^^10^^,^^36^ A study in Saudi Arabia showed an increase in fried food and soft drink consumption during the pandemic.^10^ One study in Qatar assessed the impact of COVID-19-related home confinement measures on the diet of children between 5 and 12 years of age that included 144 participants, which showed a significant increase in the total number of main meals per day, with higher consumption of unhealthy food.^37^ Accumulating evidence has shown the adverse impact of lockdown measures, and school closures on the mental health of children and adolescents and how it contributed to increased anxiety, depression, loneliness, stress, sadness, and frustration in young people and children.^38^^,^^39^ Positive associations between stressful events, emotional problems, and the consumption of fat- and sugar-dense food among children have been proven.^40^ Emotional eating, which is a maladaptive coping strategy in which people tend to consume energy-dense food products to relieve negative emotions, has been reported among children and adolescents during the COVID-19 pandemic, and it is considered a risk factor for obesity due to the consumption of high-caloric products.^41^^,^^42^ A recently published multinational study that investigated the impact of the COVID-19 pandemic on Middle Eastern Arab children’s eating habits and included children from Bahrain, Jordan, Lebanon, Saudi Arabia, United Arab Emirates, Iraq and Oman showed an increase in emotional eating among children from 72% before the pandemic to 91.5% after.^43^

We found a significant increase in the BAZ scores by 0.30 over the period of school closures consistent with previous studies.^6^^,^^31^ The increased BAZ scores translated into an increased prevalence of obesity and overweight in our sample, which increased collectively from 39% before closure to ~50% as assessed after the reopening of schools. A study in Korea reported that the BAZ of children between 4 and 14 years increased significantly by 0.22 in the COVID-19 period compared to the pre-COVID-19 period, and the proportion of overweight or obese children increased from 23.9% in the pre-COVID-19 period to 31.4% in the COVID-19 period.^44^ In Jordan, a significant increase in BAZ from 0.32 ± 1.9 before the pandemic to 0.82 ± 1.9 during the lockdown was reported among children.^6^ Emerging evidence is showing that school closures can provoke obesogenic behaviors (physical inactivity, sedentary behaviors, unhealthy diet and excessive sleep) as recent studies showed an accelerated weight gain of children after returning from the summer vacation compared to during the school year.^45^^,^^46^ Such behaviors become regulated when the children are exposed to a structured day (school day), which is known as the ‘Structured Days Hypothesis’.^47^ The hypothesis was built on the fundamental differences between the summer day and the school day. Unlike the summer day, the school day can be viewed as a structured, purposive, less autonomous and more supervised environment with compulsory components.^47^ Consequently, children with a greater sense of autonomy (e.g. during summertime) have a greater sense of choice negatively impacting their lifestyle behaviors. It is expected that there will be no room for unfavorable activities if your time is filled with favorable ones as in the school day. We can look at the COVID-19-related school closures as a 2-year summer vacation that brought a lot of unhealthy behaviors to the lives of children and adolescents. The Structured Days Theory can also explain the significant reduction of physical activity and the significant increase in screen time during school closures since most children are provided with both intentional (e.g. physical education, before/after school programs, organized sports programs) and unintentional physical activity opportunities (e.g. transitions between activities, walking to school) during a typical school day. Conversely, on an unstructured off day, children might be exposed to a prolonged period of unsupervised time when they are free to engage in unfavorable activities such as excessive digital device use.^47^ Moreover, the regularity in sleep patterns is usually lost during off days with many children going to bed late and waking up late leading to increased risks of obesity.^48^

### What this study adds

This research provides crucial insights into the impact of COVID-19 school closures on the lifestyle and weight of children and adolescents in Qatar, highlighting significant shifts in dietary habits, physical activity levels, screen time and BAZ scores. The study highlights the emergence of increased obesity and overweight prevalence among this demographic during the pandemic in Qatar. It offers a comprehensive quantitative assessment of how lifestyle behaviors among children and adolescents have been altered due to the pandemic, specifically in the context of a Middle Eastern country, and establishes a clear connection between altered lifestyle behaviors and an increase in BAZ scores. It lays the groundwork for future investigations to determine if these adverse lifestyle changes have persisted post-pandemic.

In light of our findings, we propose several strategies to mitigate the pandemic’s negative impact on children’s lifestyles. At the family level, educational programs should emphasize structured physical activities, healthy dietary habits and regulated screen time. Schools should involve parents in health-promoting activities and enhance nutrition literacy. In case of future school closures, online physical education and nutrition education should be provided, along with limiting virtual classroom screen time. At the government and policy level, national guidelines for physical activity and nutrition should be developed and disseminated, with a focus on maintaining healthy lifestyles during emergencies. The healthcare system should offer comprehensive weight management services and lifestyle counseling. Finally, researchers are encouraged to continue investigating the pandemic’s long-term lifestyle impacts and explore the factors driving these changes.

#### Limitations of the study

The strength of this study mainly lies in the objective assessment of the impact of COVID-19-related school closures on the lifestyle of children and adolescents by assessing the changes in BAZ scores before and after school closures. This study was conducted at a national level with an adequate sample size and appropriate sampling technique that facilitates the generalization of the results to students of the same age groups. We derived the sample from a trusted credible electronic health record system. It is one of the few studies conducted in the Middle East that assesses the adverse lifestyle changes among an important segment of the population (children and adolescents). On the other hand, we acknowledge some limitations. We relied on the retrospective collection of parent-reported changes in lifestyle behaviors (diet, physical activity and screen time), which might have introduced recall bias. We only included students at governmental schools as those schools are covered by the national annual growth monitoring. Moreover, the absence of historical BAZ scores data for a comparable period prior to the pandemic restricts our ability to establish a baseline against which the pandemic’s impact on BAZ scores can be accurately measured. Without pre-pandemic BAZ scores, it is challenging to definitively attribute the observed changes in BAZ scores solely to the pandemic’s influence. Lastly, the demographic characteristics of the children and adolescents in our study are representative of our study region but may not reflect the diversity found in other countries or regions.

## Conclusion

The school closures during the COVID-19 pandemic had deleterious implications for the lifestyle and weight of children and adolescents in Qatar. We found significant adverse dietary changes, a significant reduction in physical activity, a significant increase in screen time, and a significant increase in the BAZ scores that translated into an increased prevalence of obesity and overweight. After restrictive measures were eased after the pandemic, researchers need to investigate and understand whether such adverse lifestyle changes persist. To implement effective lifestyle-related interventions, Qatar’s School Health Services and Programs must collaborate and work with policymakers and other stakeholders such as students, parents and teachers at government schools.

## Supplementary Material

English_version_of_the_quesitonnaire_fdad287

the_Arabic_version_of_the_questionnaire_fdad287

## Data Availability

Data will be available upon reasonable request from the corresponding author.

## Notes

**Muna Abed Alah**, MD, Community Medicine Specialist

**Sami Abdeen**, MD

**Iheb Bougmiza**, Consultant Community Medicine

**Nagah Selim**, Senior Consultant Community Medicine
