# Supplementary material for: COVID-19 lockdowns weigh heavily on youth: an analysis of the impact on BMI for Age Z scores in children and adolescents
Source: J Public Health (Oxf). 2024 Jan 30;46(2):209–22. doi: 10.1093/pubmed/fdad287 (PMC11141597; doi:10.1093/pubmed/fdad287)
Supplement: English_version_of_the_quesitonnaire_fdad287 [file english_version_of_the_quesitonnaire_fdad287.docx]

**Supplementary Material 1: The English version of the questionnaire**

| **Assessing dietary behavioral changes over the period of school closures** | | | | | | | | | | | | | | |
| --- | --- | --- | --- | --- | --- | --- | --- | --- | --- | --- | --- | --- | --- | --- |
| 1. How many servings of fruit did your child usually eat in a day? (**A one servings of fruits equals to 1 medium fruit (e.g., banana, apple, orange), or 2 small (Kiwi, apricot) 1 cup sliced fruit** **(e.g., watermelon) or canned fruit, or ½ cup 100% fresh juice, or ¼ cup dried fruit)** *Before school closure* | Less than 1 serving/day | | | 1-2 servings per day | | | more than 2 servings /day | | | | | | | |
| 1. How many servings of fruit did your child usually eat in a day? *During school closure* | Less than 1 serving/day | | | 1-2 servings per day | | | more than 2 servings /day | | | | | | | |
| 1. How many servings of vegetables did your child usually eat in a day? **A one servings of vegetables equals to ½ cup of cooked vegetables or 1 cup of salad vegetables (raw green leafy vegetables) or ½ medium of starchy vegetables (potato) or 1 medium vegetables (cucumber, carrot).** *Before school closure* | Less than 1 serving/day. | | | 1-3 servings/day | | | 4-6 servings/day | | | More than 6 servings /day). | | | | |
| 1. How many servings of vegetables did your child usually eat in a day? *During school closure* | Less than 1 serving/day. | | | 1-3 servings/day | | | 4-6 servings/day | | | More than 6 servings /day). | | | | |
| 1. How much soft drinks, sweetened beverages, energy drinks or sports drinks did your child usually drink? (**One can of soft drink = 1 ½ cups)** *Before school closure* | My child does not drink these drinks | | < 1 cup/week | | | 1-3 cups/week | | 4-6 cups/week | | | 1-2 cups/day | | 3 or more cups/day | |
| 1. How much soft drinks, sweetened beverages, energy drinks or sports drinks did your child usually drink? (*During school closure* | My child does not drink these drinks | | < 1 cup/week | | | 1-3 cups/week | | 4-6 cups/week | | | 1-2 cups/day | | 3 or more cups/day | |
| 1. How often did your child eat fried food (French-fried potatoes, fried chicken….) prepared at home? *Before school closure* | never or rarely | less than once a week | | | About 1 to 2 times a week | | About 3 to 4 times a week | | About 5 to 6 times a week | | | about once a day | | 2 or more times a day |
| 1. How often did your child eat fried food (French-fried potatoes, fried chicken….) prepared at home? *During school closure* | never or rarely | less than once a week | | | About 1 to 2 times a week | | About 3 to 4 times a week | | About 5 to 6 times a week | | | about once a day | | 2 or more times a day |
| 1. How often did your child eat junk food (burger, pizza…) from fast food restaurants? *Before school closure* | never or rarely | less than once a week | | | About 1 to 2 times a week | | About 3 to 4 times a week | | About 5 to 6 times a week | | | about once a day | | 2 or more times a day |
| 1. How often did your child eat junk food (burger, pizza…) from fast food restaurants? *During school closure* | never or rarely | less than once a week | | | About 1 to 2 times a week | | About 3 to 4 times a week | | About 5 to 6 times a week | | | about once a day | | 2 or more times a day |
| 1. How often did your child eat sugar-based sweets/ sweets (candies, chocolate, jam, Nutella...)? *Before school closure* | never or rarely | less than once a week | | | About 1 to 2 times a week | | About 3 to 4 times a week | | About 5 to 6 times a week | | | about once a day | | 2 or more times a day |
| 1. How often did your child eat sugar-based sweets/ sweets (candies, chocolate, jam) *During school closure* | never or rarely | less than once a week | | | About 1 to 2 times a week | | About 3 to 4 times a week | | About 5 to 6 times a week | | | about once a day | | 2 or more times a day |

| **Assessing physical activity changes over the period of school closures** | | | | | | | | |
| --- | --- | --- | --- | --- | --- | --- | --- | --- |
| 1. In a typical week, on how many days have your child done a total of 60 minutes or more of physical activity, which was enough to raise his/her breathing rate? This may include sport, exercise and brisk walking, cycling for recreation or to get to and from places or active playing’ (*Before school closure.)* | 0 | 1 | 2 | 3 | 4 | 5 | 6 | 7 |
| 1. In a typical week, on how many days have your child done a total of 60 minutes or more of physical activity, which was enough to raise his/her breathing rate? This may include sport, exercise and brisk walking, cycling for recreation or to get to and from places or active playing’ (*During school closure.)* | 0 | 1 | 2 | 3 | 4 | 5 | 6 | 7 |
| 1. Did your child use to practice any type of sports on regular bases outside the school? *Before school closure.* | Yes | | | | No | | | |
| 1. Did your child use to practice any type of sports on regular bases outside the school? *During school closure.* | Yes | | | | No | | | |

| **Assessing screen time before and during school closures** | | |
| --- | --- | --- |
| 1. How many hours did your child use to use digital devices in a day (excluding time spent for online school classes)? *Before school closure* | During Weekdays |  |
| 1. How many hours did your child use to use digital devices in a day (excluding time spent for online school classes)? *Before school closure* | During Weekend |  |
| 1. How many hours did your child use to use digital devices in a day (excluding time spent for online school classes)? *During school closure* | During Weekdays |  |
| 1. How many hours did your child use to use digital devices in a day (excluding time spent for online school classes)? *During school closure* | During Weekend |  |
|  |  |  |
