# Supplementary material for: COVID-19 lockdowns weigh heavily on youth: an analysis of the impact on BMI for Age Z scores in children and adolescents
Source: J Public Health (Oxf). 2024 Jan 30;46(2):209–22. doi: 10.1093/pubmed/fdad287 (PMC11141597; doi:10.1093/pubmed/fdad287)
Supplement: the_Arabic_version_of_the_questionnaire_fdad287 [file the_arabic_version_of_the_questionnaire_fdad287.docx]

**Supplementary material 2: The Arabic version of the questionnaire**

| **تقييم التغيرات في النظام الغذائي خلال فترة اغلاق المدارس** | | | | | | | | |
| --- | --- | --- | --- | --- | --- | --- | --- | --- |
| اكثر من 2 حصة في اليوم | | | | 1-2 حصة في اليوم | | أقل من حصة في اليوم | | 1. كم حصة من الفاكهة كان يأكل طفلك عادة في اليوم (**حصة واحدة من الفاكهة تعادل ثمرة فاكهة متوسطة الحجم (مثل الموز أو التفاح أو البرتقال) او ثمرتي فاكهة صغيرة الحجم (مثل الكيوي او المشمش) او 1 كوب فاكهة مقطعة (مثل البطيخ )أو الفاكهة المعلبة او 1/2 كوب 100% عصير طبيعي أو 1/4 كوب فاكهة مجففة) قبل إغلاق المدرسة** |
| اكثر من 2 حصة في اليوم | | | | 1-2 حصة في اليوم | | أقل من حصة في اليوم | | 1. كم حصة من الفاكهة كان يأكل طفلك عادة في اليوم ؟ أثناء إغلاق المدرسة |
| أكثر من 6 حصص في اليوم | | 4-6 حصة في اليوم | | 1-3 حصة في اليوم | | أقل من حصة في اليوم | | 1. كم حصة من الخضار كان يأكل طفلك عادة في اليوم؟ (**تعادل حصة واحدة من الخضار نصف كوب من الخضار المطبوخة أو 1 كوب من خضروات السلطة (الخضروات الورقية الخضراء النيئة) او ½ ثمرة من الخضار الغني بالنشا متوسط الحجم (البطاطا) او 1 ثمرة من الخضار متوسط الحجم (خيار, جزر)** قبل إغلاق المدرسة |
| أكثر من 6 حصص في اليوم | | 4-6 حصة في اليوم | | 1-3 حصة في اليوم | | أقل من حصة في اليوم | | 1. كم حصة من الخضار كان يأكل طفلك عادة في اليوم؟ أثناء إغلاق المدرسة |
| 3 أو أكثر  كوب /يوم | 1-2 كوب/ اليوم | | 4-6 كوب/  الأسبو ع | 1-3 كوب/  الأسبو ع | < 1 كوب/  الأسبو ع | | طفلي لا يشرب هذه المشروبات | 1. ما هي كمية المشروبات الغازية أو العصائر المحلاة أو مشروبات الطاقة أو المشروبات الرياضية التي اعتاد طفلك أن يشربها (علبة واحدة من المشروبات الغازية = كوب و نصف) قبل إغلاق المدرسة؟ |

| 3 أو أكثر  كوب /يوم | | 1-2 كوب/ اليوم | | 4-6 كوب/  الأسبو ع | | 1-3 كوب/  الأسبو ع | | < 1 كوب/  الأسبو ع | | طفلي لا يشرب هذه المشروبات | | 1. ما هي كمية المشروبات الغازية أو العصائر المحلاة أو مشروبات الطاقة أو المشروبات الرياضية التي اعتاد طفلك أن يشربها (علبة واحدة من المشروبات الغازية = كوب و نصف) أثناء إغلاق المدرسة؟ |
| --- | --- | --- | --- | --- | --- | --- | --- | --- | --- | --- | --- | --- |
| مرتين أو أكثر في اليوم | حوالي مرة في اليوم | | حوالي 5  إلى 6 مرات في الأسبو ع | | حوالي 3  إلى 4 مرات في الأسبو ع | | حوالي مرة إلى مرتين في الأِسبو ع | | أقل من مرة في الأسبو ع | | بتاتًا أو  نادرًا | 1. كم مرة اعتاد طفلك أن يتناول طعامًا مقليًا محضراً في المنزل (بطاطس مقلية، أو دجاج مقلي....) ؟ قبل إغلاق المدرسة |
| مرتين أو أكثر في اليوم | حوالي مرة في اليوم | | حوالي 5  إلى 6 مرات في الأسبو ع | | حوالي 3  إلى 4 مرات في الأسبو ع | | حوالي مرة إلى مرتين في الأِسبو ع | | أقل من مرة في الأسبو ع | | بتاتًا أو  نادرًا | 1. كم مرة اعتاد طفلك أن يتناول طعامًا مقليًا محضراً في المنزل (بطاطس مقلية، أو دجاج مقلي....) ؟ أثناء إغلاق المدرسة |
| مرتين أو أكثر في اليوم | حوالي مرة في اليوم | | حوالي 5  إلى 6 مرات في الأسبو ع | | حوالي 3  إلى 4 مرات في الأسبو ع | | حوالي مرة إلى مرتين في الأِسبو ع | | أقل من مرة في الأسبو ع | | بتاتًا أو  نادرًا | 1. كم مرة اعتاد طفلك أن يتناول الوجبات السريعة (برجر، بيتزا ...) من مطاعم الوجبات السريعة؟ قبل إغلاق المدرسة |
| مرتين أو أكثر في اليوم | حوالي مرة في اليوم | | حوالي 5  إلى 6 مرات في الأسبو ع | | حوالي 3  إلى 4 مرات في الأسبو ع | | حوالي مرة إلى مرتين في الأِسبو ع | | أقل من مرة في الأسبو ع | | بتاتًا أو  نادرًا | 1. كم مرة اعتاد طفلك أن يتناول الوجبات السريعة (برجر،بيتزا...) من مطاعم الوجبات السريعة؟ أثناء إغلاق المدرسة |
| مرتين أو أكثر في اليوم | حوالي مرة في اليوم | | حوالي 5  إلى 6 مرات في الأسبو ع | | حوالي 3  إلى 4 مرات في الأسبو ع | | حوالي مرة إلى مرتين في الأِسبو ع | | أقل من مرة في الأسبو ع | | بتاتًا أو  نادرًا | 1. كم مرة اعتاد طفلك أن يتناول الاطعمة الغنية بالسكر والحلويات مثل السكاكر، الشوكولاته، المربى؟ قبل إغلاق المدرسة |
| مرتين أو أكثر في اليوم | حوالي مرة في اليوم | | حوالي 5  إلى 6 مرات في الأسبو ع | | حوالي 3  إلى 4 مرات في الأسبو ع | | حوالي مرة إلى مرتين في الأِسبو ع | | أقل من مرة في الأسبو ع | | بتاتًا أو  نادرًا | 1. كم مرة اعتاد طفلك أن يتناول الاطعمة الغنية بالسكر والحلويات مثل السكاكر، الشوكولاته، المربى، النوتيلا... ؟ أثناء إغلاق المدرسة |

| **تقييم التغيرات في النشاط البدني خلال فترة اغلاق المدارس** | | | | | | | | | | |
| --- | --- | --- | --- | --- | --- | --- | --- | --- | --- | --- |
| 7 | 6 | 5 | | 4 | 3 | 2 | 1 | | 0 | 1. في الأسبوع ،كم عدد الأيام التي اعتاد فيها طفلك أن يمارس ما مجموعه 60 دقيقة أو أكثر من النشاط البدني والذي كان كافياً لرفع معدل تنفسه؟ (قد يشمل ذلك الرياضة أوالتمارين أوالمشي السريع أو ركوب الدراجات للاستجمام أو للوصول من وإلى الأماكن او اللعب النشط) قبل إغلاق المدرسة. |
| 7 | 6 |  | 5 | 4 | 3 | 2 |  | 1 | 0 | 1. في الأسبوع ،كم عدد الأيام التي اعتاد فيها طفلك أن يمارس ما مجموعه 60 دقيقة أو أكثر من النشاط البدني والذي كان كافياً لرفع معدل تنفسه؟ (قد يشمل ذلك الرياضة أو التمارين أو المشي السريع أو ركوب الدراجات للاستجمام أو للوصول من وإلى الأماكن او اللعب النشط) أثناء إغلاق المدرسة |
| لا | | | | | نعم | | | | | 1. هل اعتاد طفلك الذهاب على ممارسة أي نوع من الرياضات بانتظام خارج نطاق المدرسة؟ قبل إغلاق المدرسة. |
| لا | | | | | نعم | | | | | 1. هل اعتاد طفلك على ممارسة أي نوع من الرياضات بانتظام خارج نطاق المدرسة؟ أثناء إغلاق المدرسة. |

| **الوقت الممضي أمام الشاشات الرقمية قبل و أثناء اغلاق المدارس** | | |
| --- | --- | --- |
|  | خلال أيام الأسبوع | 1. ما إجمالي عدد الساعات التي اعتاد طفلك أن يقضيها في استخدام الأجهزة الرقمية في اليوم (باستثناء الوقت المخصص للتعليم عن بعد و مشاهدة التلفاز) قبل إغلاق المدرسة ؟ |
|  | خلال عطلة نهاية الأسبوع | 1. ما إجمالي عدد الساعات التي اعتاد طفلك أن يقضيها في استخدام الأجهزة الرقمية في اليوم (باستثناء الوقت المخصص للتعليم عن بعد و مشاهدة التلفاز) قبل إغلاق المدرسة |
|  | خلال أيام الأسبوع | 1. ما إجمالي عدد الساعات التي اعتاد طفلك أن يقضيها في استخدام الأجهزة الرقمية في اليوم (باستثناء الوقت المخصص للتعليم عن بعد (أثناء إغلاق المدرسة |
|  | خلال عطلة نهاية الأسبوع | 1. ما إجمالي عدد الساعات التي اعتاد طفلك أن يقضيها في استخدام الأجهزة الرقمية في اليوم (باستثناء الوقت المخصص للتعليم عن بعد أثناء إغلاق المدرسة ؟ |
